# Plasma chromogranin A is a marker of death in elderly patients presenting with symptoms of heart failure

**DOI:** 10.1530/EC-14-0017

**Published:** 2014-03-15

**Authors:** Jens P Goetze, Linda M Hilsted, Jens F Rehfeld, Urban Alehagen

**Affiliations:** 1 Department of Clinical Biochemistry, Rigshospitalet University of Copenhagen Copenhagen Denmark; 2 Division of Cardiovascular Medicine, Department of Medicine and Health Sciences, Faculty of Health Sciences, Department of Cardiology UHL, County Council of Östergötland Linköping University Linköping Sweden

## Abstract

Cardiovascular risk assessment remains difficult in elderly patients. We examined whether chromogranin A (CgA) measurement in plasma may be valuable in assessing risk of death in elderly patients with symptoms of heart failure in a primary care setting. A total of 470 patients (mean age 73 years) were followed for 10 years. For CgA plasma measurement, we used a two-step method including a screening test and a confirmative test with plasma pre-treatment with trypsin. Cox multivariable proportional regression and receiver-operating curve (ROC) analyses were used to assess mortality risk. Assessment of cardiovascular mortality during the first 3 years of observation showed that CgA measurement contained useful information with a hazard ratio (HR) of 5.4 (95% CI 1.7–16.4) (CgA confirm). In a multivariate setting, the corresponding HR was 5.9 (95% CI 1.8–19.1). When adding N-terminal proBNP (NT-proBNP) to the model, CgA confirm still possessed prognostic information (HR: 6.1; 95% CI 1.8–20.7). The result for predicting all-cause mortality displayed the same pattern. ROC analyses in comparison to NT-proBNP to identify patients on top of clinical variables at risk of cardiovascular death within 5 years of follow-up showed significant additive value of CgA confirm measurements compared with NT-proBNP and clinical variables. CgA measurement in the plasma of elderly patients with symptoms of heart failure can identify those at increased risk of short- and long-term mortality.

## Introduction

Heart failure is a syndrome comprising cardiac dysfunction and neurohumoral activation. Medical treatment aims at neutralizing hormonal actions by blocking receptors or inhibiting activation of vasoconstrictive substances. In particular, blockade of the concomitant sympathetic activation via adrenergic receptors has reduced mortality and morbidity in chronic heart failure patients.

Plasma adrenalin and noradrenalin are dominantly secreted from the adrenal glands. Another substance secreted from the adrenal medulla is chromogranin A, or CgA (1). CgA measurement in plasma has a central role in the diagnosis and treatment follow-up of neuroendocrine tumors, since these tumors often produce vast amounts of CgA (2, 3, 4). In the cardiological setting, CgA measurement has only been explored in small patient cohorts with acute coronary syndrome or heart failure (5, 6, 7, 8, 9). Interestingly, a few reports have suggested that the heart muscle itself produces CgA that may contribute to the plasma pool (10, 11). For now, CgA plasma measurement as a biomarker in heart failure is still only examined in highly selected patients and cannot be recommended for general use (12). Moreover, methodological problems on CgA measurement have hampered the general use of CgA as a biomarker, because CgA processing is extensive, variable, and harbors a plethora of fragments.

In the present study, we examined a cohort of elderly patients with symptoms suggestive of heart failure, i.e. tiredness, dyspnea, and/or edema, in the primary care setting. For CgA plasma measurement, we used state-of-the-art analyses (13) that measure a well-defined epitope in the CgA protein (screen test), and an extended version of the analysis that quantitates the total amount of CgA products in blood irrespective of post-translational processing (confirm test).

## Subjects and methods

The design of the study has previously been published (14, 15). The patient population consisted of patients, 65–87 years of age, recruited from a primary health center with symptoms of heart failure (dyspnea, tiredness, and/or peripheral edema). A cardiologist reviewed all records of patients with the listed symptoms and then met all patients, performed a clinical examination and a 2D echocardiography, and established new patient records. The revised illnesses in the patients have also been reported previously (16). Patients in whom heart failure could not be excluded were invited to participate in the study. All participants were included in 1996 and were followed for 13 years. The evaluation of the markers, however, covered a follow-up period of 10 years based on blood samples collected at study inclusion. During the follow-up period, all mortality was registered and data were recorded from death certificates or autopsy records. Written, informed consent was obtained from all patients at inclusion, and the study protocol was approved by the Regional Ethical Review Board in Linköping.

### Echocardiography

Doppler echocardiographic examinations (Accuson XP-128c) were performed with participants in the left lateral position. Normal left ventricular systolic function was defined as EF ≥50%: severely impaired systolic function was defined as EF *<*30% (17, 18). For assessment of diastolic function, mitral flow E:A ratios and pulmonary venous flow patterns were analyzed and compared with age-adjusted reference values.

### Biochemical analyses

N-terminal proBNP (NT-proBNP or proBNP 1–76) was measured on the Elecsys 2010 platform (Roche Diagnostics). This assay uses two polyclonal antibodies directed against amino acid sequences 1–21 and 39–50 respectively. Total assay CV was 4.8% at 220 ng/l and 2.1% at 4254 ng/l (*n*=70).

CgA was measured with two assays. First, an in-house immunoassay using antibodies raised against the 340–348 CgA fragment was used as screen test. Plasma was then incubated with trypsin to cleave CgA and CgA fragments at dibasic cleavage sites (13). Moreover, this enzymatic treatment removes possible interference from plasma proteins (19, 20). The same monospecific RIA was then employed again and thus quantitated the total CgA concentration in plasma. The extended test was used as a confirmatory test in the present study. This assay has previously been employed successfully for the diagnosis and follow-up of carcinoid tumors (21). The interassay precision at 60 pmol/l is <20% (JP Goetze, LM Hilsted, JF Rehfeld & U Alehagen, 2013, unpublished observations).

### Statistical analyses

Descriptive data are presented as percentages or mean and s.d. In the case of continuous variables, comparative analyses were performed using the Student's unpaired two-sided *t*-test, whereas the *χ* ^2^ test was used for discrete variables. Cox proportional hazard regression analyses as well as a Kaplan–Meier analysis were used to analyze the risk of mortality during the follow-up period. Censored patients were those still alive at end of the study period or who had died of other causes than cardiovascular disease. Completed patients comprised those who had died due to cardiovascular disease.

The assumption of proportionality was tested as long follow-up. As the assumption was not fulfilled, we chose to present mortality data in two steps: short-term mortality with a follow-up time of 3 years and long-term mortality with a follow-up time of up to 10 years. To evaluate the possible additive prognostic effects of the biomarkers in multivariate analysis, Cox proportional regression analyses were carried out. Three different models have been used: model I consisted of a univariate evaluation, model II consisted of a multivariate evaluation in which clinical variables that could influence the risk of cardiovascular mortality were included. In model III, all clinical variables from model II were included and NT-proBNP was added to the model.

In order to evaluate specificity and sensitivity, receiver-operating curve (ROC) analyses have been performed by using the method advised by DeLong *et al*. (22). To evaluate a possible significant difference between two ROC curves, the contrast between them has been evaluated. A clinical prognostic index was developed based on the β coefficients obtained from the Cox proportional hazard regression analyses. Three models were tested in the ROC analyses: model I, clinical variables only; model II, clinical variables+NT-proBNP, and model III, clinical variables+NT-proBNP+CgA confirm. The clinical variables included were as follows: NYHA functional class III, Hb <120 g/l, diabetes, male gender, and ischemic heart disease. The area under curve was calculated for the three models. For model I, a sensitivity/specificity of 56 and 85% was chosen; for model II, the corresponding values were 71 and 77%; and finally for model III, the corresponding values were 73 and 76%. A *P* value <0.05 was considered statistically significant. All data were analyzed using standard software packages (Statistica v. 12.0, Statsoft, Inc., Analyse-it v.3.53; Analyse-it Software Ltd, Tulsa, OK, USA).

## Results

An elderly population with a mean age of 73 years and with an equal distribution between males/females was evaluated. The basic characteristics are presented in Supplementary Table 1, see section on supplementary data given at the end of this article. All patients were presented with dyspnea, tiredness, and/or peripheral edema. The study population was followed for 13 years (median 4725 days, range 242–5112). The first patient was included in January 1996, and the last follow-up date was December 31, 2009. As patients in treatment with proton pump inhibitors display increased plasma concentrations of CgA due to increased production of CgA from gastric enterochromaffin-like cells, it is important to identify patients with such treatment. In our population, only a small fraction was on treatment with proton pump inhibitors (*n*=3; 0.6%) and none of the three had high levels of CgA. These patients were, therefore, included in the calculations.

During the observation period, 226 patients (48%) suffered all-cause mortality and 146 patients (31%) cardiovascular mortality. Notably, no patient was lost during follow-up. The part that survived all-cause mortality during the follow-up period had a median observation period of 4923 days (range 4773–5112), whereas those that did not survive during the follow-up period had a median observation period of 2723 days (range 242–5018). The distribution of cardiovascular mortality in the different quartiles of proBNP and CgA screen and confirm is shown in Supplementary Table 2, see section on supplementary data given at the end of this article. We chose to use quartiles in order to apply more than one cutoff value, as for example by using medians. An increase in cardiovascular mortality was found as a function of increasing quartiles. A similar pattern, although different in sizes, was seen for all biomarkers. The greatest difference in mortality between the quartiles could, however, be seen in the NT-proBNP biomarker. In the cardiovascular mortality evaluation over 10 years, the difference in mortality between 1st and 4th quartiles in CgA confirm test was 24.2%, whereas the difference in plasma NT-proBNP concentration was 41.9%. Smaller differences between 1st and 4th quartiles were found with the CgA screen test.

### Prognostic information

When assessing the prognostic information regarding all-cause mortality during the first 3 years of observation, we found (model I; univariate regression) that CgA confirm testing contained significant prognostic information with a hazard ratio (HR) of 3.49 (95% CI 1.51–8.10) as shown in Supplementary Table 3, see section on supplementary data given at the end of this article. In model II, where clinical variables were added to the model, the CgA confirm test still contained information with HR of 3.48 (95% CI 1.42–8.53). After adding the gold standard biomarker for heart failure (NT-proBNP) into the model (model III), the CgA confirm test still exhibited significant and independent prognostic information (HR 3.61, 95% CI 1.42–9.15). The CgA screen test did not, however, exhibit any significant information in this setup. Analysis for cardiovascular mortality during the same time frame showed a similar pattern, i.e., significant HRs for the CgA confirm test in models I, II, and III (shown in Table 1). The HRs were even higher, but the CIs were also wider due to small samples. Analyzing the prognostic information for all-cause mortality in the time frame up to 10 years, CgA screen and confirm testing exhibited significant prognostic information in model I (Table 2). In the multivariate setting in model II, only CgA confirm test could exhibit significant prognostic information, which persisted also in competition with NT-proBNP in model III (HR 1.44, 95% CI 1.05–1.98). Finally, when analyzing the cardiovascular mortality during the same time frame (Table 3), it was noted that both the CgA screen and confirm measurements reached significant prognostic information in model I, and that CgA confirm measurements reached significant prognostic information both in model II and III (HR 1.77, 95% CI 1.21–2.60, and HR 1.66, 95% CI 1.13–2.45 respectively).

**Figure 1:**
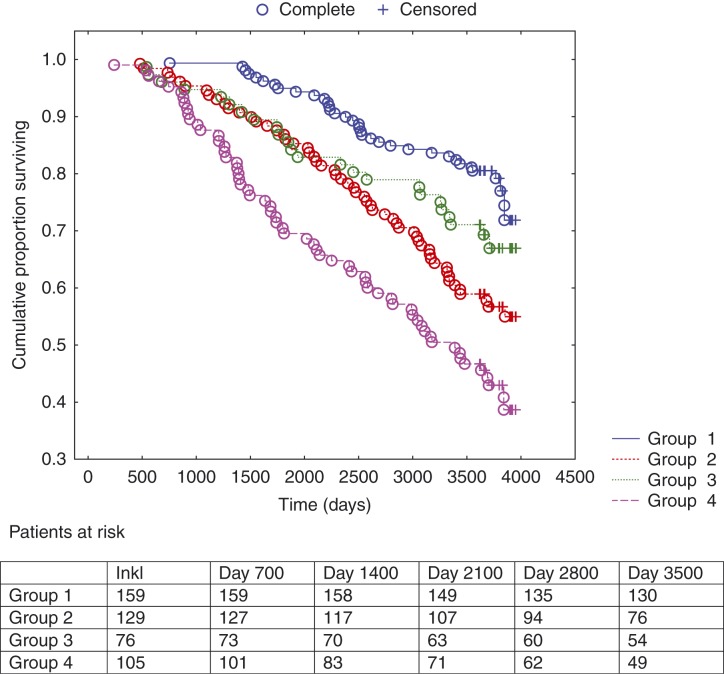
Kaplan–Meier analysis illustrating the distribution of all-cause mortality expressed as different combinations of level above vs below median concentration of chromogranin A confirm test and NT-proBNP in the study population during 10 years of follow-up. Censored patients were patients still alive at end of the study period. Completed patients were patients who had died due to all-cause mortality. Group 1: NT-proBNP <median+chromogranin A confirm<median; Group 2: NT-proBNP>median+chromogranin A confirm<median; Group 3: NT-proBNP<median+chromogranin A confirm>median; and Group 4: NT-proBNP>median+chromogranin A confirm>median.

**Figure 2:**
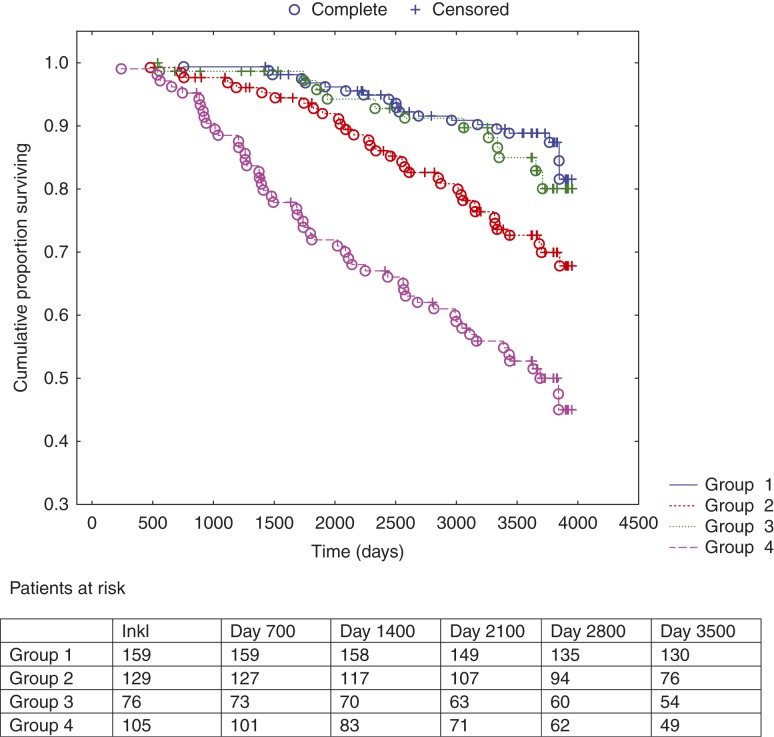
Kaplan–Meier analysis illustrating the distribution of cardiovascular mortality expressed as different combinations of level above vs below median concentration of chromogranin A confirm test and NT-proBNP in the study population during 10 years of follow-up. Censored patients were patients still alive at end of the study period or who had died of other reasons than cardiovascular disease. Completed patients were patients who had died due to cardiovascular mortality. Group 1: NT-proBNP <median+chromogranin A confirm< median; Group 2: NT-proBNP>median+chromogranin A confirm<median; Group 3: NT-proBNP<median+chromogranin A confirm>median; and Group 4: proBNP>median+chromogranin A confirm>median.

Analyzing the study population by Kaplan–Meier analyses regarding all-cause mortality during 10 years of follow-up distributed in different combinations of groups of CgA confirm test and NT-proBNP when applying the median concentration as cut-point, we found that an increasing number of patients did not survive as the plasma concentration increased, fewest in group 4 where both biomarkers were above the cut-point (Fig. 1). Also, only ∼40% of the patients in that group were still alive after 10 years. Figure 2 shows the cardiovascular mortality during 10 years of follow-up distributed in different combinations of groups of CgA confirm test and NT-proBNP when applying the median concentration as cut-point; an increase in mortality between the groups 3 and 4 in relation to groups 1 and 2 could be seen and most clearly in group 4, where both the plasma markers were above the cut-point. In this group, only ∼45% were still alive after 10 years.

**Figure 3:**
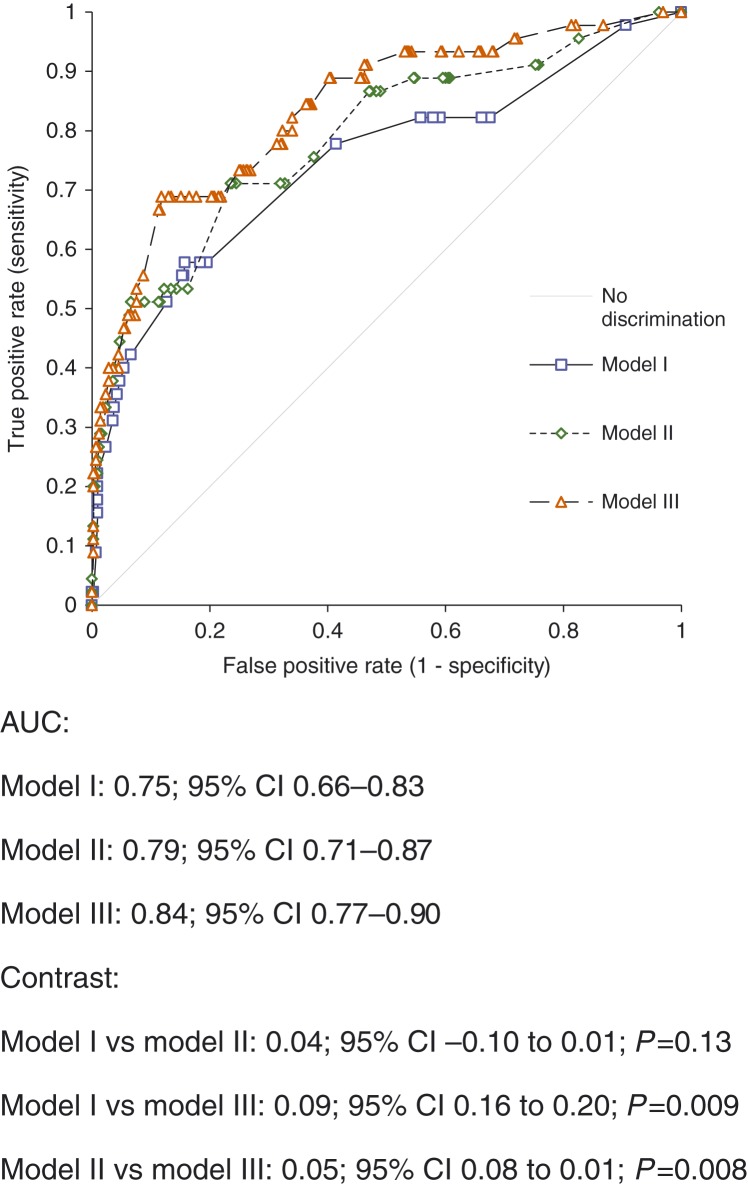
ROC analysis illustrating the ability of chromogranin A screen, chromogranin A confirm and NT-proBNP to identify those at risk of cardiovascular death within an elderly primary health care population during 3 years of follow-up.

In all multivariate evaluations including NT-proBNP in the setting (model III), the CgA confirm test contained significant and independent prognostic information besides that of the other variables, including NT-proBNP, the standard biomarker for heart failure. Finally, ROC curve analyses of the ability of CgA measurements in comparison to NT-proBNP on top of some well-known clinical variables were performed in three models. In model I, only clinical variables were performed to identify patients who are at risk of cardiovascular death within the 3 years of follow-up (Fig. 3). In model II, NT-proBNP was added to the clinical variables, and finally in model III, CgA confirm was added to clinical variables+NT-proBNP. From the AUC analyses, a significant additive prognostic information could be obtained by adding CgA confirm measurements on top of NT-proBNP (AUC: 0.84, 95% CI 0.77–0.90 vs AUC: 0.79, 95% CI 0.71–0.87). The difference in AUC between model II and III was significant, *P*=0.008. In order to evaluate what this information could result in, we analyzed all patients that died within 5 years of cardiovascular death. Applying model I, 25/45 (56%) could be identified. Applying model II, 32/45 (71%) of the deceased patients could be identified: if applying model III, 36/45 (80%) of the deceased patient could be identified. The same evaluation of cardiovascular mortality on a 3-year-follow-up period gave the following information: model I 10/17 (59%), model II 12/17 (72%), and finally model III 14/17 (82%). Thus, addition of CgA was associated with a 10% increase in identifying patients at risk of cardiovascular mortality.

## Discussion

The present study shows that CgA measurement in elderly patients presenting with symptoms of heart failure can identify those at increased risk of both short- and long-term mortality. By using an assay that measures all forms of CgA in plasma, accurate quantitation could be achieved that contains information beyond a ‘gold standard’ risk marker in heart failure: thus, the data suggest for the first time that CgA plasma measurement may be a general risk marker in elderly patients. As our cohort consists of patients with unspecific symptoms suggestive of heart failure, of which only 12% reached an actual heart failure diagnosis on echocardiography, our data thus extend from former reports on selected patients with established heart failure (5, 9).

In the Cox proportional regression analyses it is clear that the CIs are wide for the CgA confirm measurements, which is why the actual figures of the HR should be interpreted with caution. We have, however, included the measurements of NT-proBNP in model III in the tables to illustrate that by use of the median plasma concentration as cut-point in this study population, even this biomarker – the most well-known biomarker in heart failure evaluations – the CIs obtained are even wider than those for the CgA confirm measurements. From the evaluation, CgA confirm evaluations have significant and independent prognostic information that goes beyond that of NT-proBNP, a finding that also was noted in the ROC analysis.

CgA measurement in cardiac patients has only been pursued in selected patient cohorts, mostly with ischemic heart disease (acute coronary syndromes) (5, 6, 7, 8, 9). In these patients, CgA measurement contained prognostic information on risk of death, which prompted us to test CgA measurement in a different cohort. Notably, the present study is based on elderly patients presenting with unspecific symptoms of heart failure in a primary setting, where most patients will be handled. Also, our study with no patients lost during the 13 years' follow-up allows us to conclude without bias. The study setup thus goes beyond the measurement of, for instance, left ventricular systolic function and is rather based on patient' common symptoms such as tiredness, dyspnea, and/or peripheral edema. We believe that such a design in itself has an important value to clinicians when employing plasma markers concerning risk. For now, we conclude that CgA plasma measurement by using the new CgA measurement assay in heart failure patients is useful as earlier suggested (12), and that the marker can even be applied in patients with symptoms suggestive of the condition.

CgA in plasma is a complex system of peptides (1). The intact CgA protein acts as a precursor that is processed before and after cellular release to multiple fragments, some of which are suggested to have biological activity (23). The many fragments severely challenge the choice of method for measurement. Moreover, measuring the selected epitope (sequence 340–348) after tryptic cleavage allows for quantitation of the total molar amount of translational products in plasma (for review, please see reference (24)). In the present report, we have used both methods as a screen and a confirm test respectively. Thus, the plasma measurements report accurately on CgA concentrations in plasma, which not all CgA assays do. In this context, we recently examined diagnostic immunoassays for a more well-defined plasma marker, gastrin (25). The results showed that more than half of the assays were inaccurate to an extent, from which the diagnosis of malignant gastrinoma was missed. We therefore recommend using validated CgA assays with precise knowledge of the measured epitope.

CgA has been shown to be produced in the heart itself (10, 11). For long, it has been known that the heart produces and secretes noradrenaline (26); in fact, the earliest hypotheses on the endocrine heart stem from this observation (27). However, the contribution of cardiac CgA to plasma is more debatable, as for instance the adrenal medulla and the gastrointestinal tract are major sites of CgA production. Our results do not answer the question of organ specificity to the measured CgA in the patient plasma, but we note that CgA measurement still proves valuable even when including proBNP measurement in the analyses. Thus, we suggest that increased CgA may dominantly represent sympathetic activity in the adrenal medulla and therefore relate to general stress in disease. However, further experimental studies are now warranted to identify the major source of the CgA in patients.

In the European Society of Cardiology Guidelines 2008 on the diagnosis and handling of heart failure patients, it is stated that in those with signs/symptoms of heart failure and NT-proBNP plasma concentrations ≤400 ng/l, the possibility of heart failure should be further considered. In the subpopulation, in which a corresponding proBNP concentration was obtained and a low risk of mortality was to be expected according to the NT-proBNP concentration, we analyzed patients with CgA measurements greater than median plasma concentration in a univariate Cox proportional hazard regression including 3 years of observation. In spite of a low NT-proBNP concentration, a significant increase in the risk of all-cause mortality was identified with CgA confirm measurement (HR 4.17, 95% CI 1.04–16.69, *P*=0.04). These data are highly encouraging. Even though the CI is wide, we suggest that CgA measurement in heart failure patients may have the largest clinical potential in ‘the grey zone’ patients with natriuretic peptide levels below the presently set diagnostic cutoff values.

### Limitations

As the study contains patients from a primary health care population, the majority do not have objective signs of impaired cardiac function in spite of prevailing symptoms in all patients. However, the cohort does represent the true population of elderly patients that general practitioners face in clinical practice. We therefore believe that the obtained information regarding CgA is interesting and applicable to a primary health care population. The evaluated population is also limited in age span, and it is thus not possible without uncertainties to extrapolate into another age group. However, the choice of age group was based on the fact that this age group is the most common among those that the general practitioner meets with the corresponding signs/symptoms.

## Supplementary data

This is linked to the online version of the paper at http://dx.doi.org/10.1530/EC-14-0017.

## Author contribution statement

Dr U Alehagen had full access to all of the data in the study and takes responsibility for the data integrity and the accuracy of the data analyses. Study concept and design: J P Goetze, U Alehagen. Design and development of CgA assays: J F Rehfeld. Acquisition of data: U Alehagen, L M Hilsted, J P Goetze. Analysis and interpretation of data: U Alehagen, J P Goetze. Drafting of the manuscript: J P Goetze, U Alehagen. Critical revision of the manuscript: U Alehagen, J P Goetze, J F Rehfeld, L M Hilsted. Statistical analysis: U Alehagen. Obtained funding: U Alehagen, J P Goetze. Study supervision: U Alehagen, J P Goetze. Financial disclosure: None to report.

## Figures and Tables

**Table 1:** Cox proportional hazard regression analysis of risk of cardiovascular mortality in three regression models during 3 years of follow-up in the study population

**Biomarker**	**Model I**			**Model II**			**Model III**		
Hazard ratio	95% CI	*P* value	Hazard ratio	95% CI	*P* value	Hazard ratio	95% CI	*P* value
Chromogranin A screen>median	0.94	0.36–2.43	0.89	0.74	0.27–2.00	0.55	0.74	0.27–2.01	0.56
Chromogranin A confirm>median	5.35	1.74–16.43	0.003	5.90	1.82–19.06	0.003	6.10	1.80–20.71	0.004
NT-proBNP>median	–	–	–	–	–	–	11.03	2.38–51.09	0.002

Model I, univariate analysis; model II, model adjusted for hypertension, ischemic heart disease, eGFR <60 ml/min, male gender, smoking habit, peripheral edema, rales, age >80 years, Hb <120 g/l; model III, all clinical variables from model II +4th quartile of NT-proBNP. Median of chromogranin A screen test: 65 pmol/l; median of chromogranin A confirm test: 531 pmol/l; median of NT-proBNP: 220 pmol/l. Each of the two biomarkers were evaluated one at a time in the different models. NT-proBNP evaluation illustrated in model III is obtained from the analysis including chromogranin A confirm test.

**Table 2:** Cox proportional hazard regression analysis of risk of all-cause mortality in three regression models during 10 years of follow-up in the study population

**Biomarker**	**Model I**			**Model II**			**Model III**		
Hazard ratio	95% CI	*P* value	Hazard ratio	95% CI	*P* value	Hazard ratio	95% CI	*P* value
Chromogranin A screen>median	1.47	1.09–1.98	0.01	1.31	0.97–1.78	0.08	1.27	0.94–1.72	0.13
Chromogranin A confirm>median	1.72	1.28–2.32	0.0003	1.50	1.09–2.05	0.01	1.44	1.05–1.98	0.02
NT-proBNP>median	–	–	–	–	–	–	2.06	1.47–2.87	<0.0001

Model I, univariate analysis; model II, model adjusted for hypertension, ischemic heart disease, eGFR <60 ml/min, male gender, smoking habit, peripheral edema, rales, age >80 years, Hb <120 g/l; model III, all clinical variables from model II +4th quartile of NT-proBNP. Median of chromogranin A screen test: 65 pmol/l; median of chromogranin A confirm test: 531 pmol/l; median of NT-proBNP: 220 pmol/l. Each of the two biomarkers has been evaluated one at a time in the different models. NT-proBNP evaluation illustrated in model III is obtained from the analysis including chromogranin A confirm test.

**Table 3:** Cox proportional hazard regression analysis of risk of cardiovascular mortality in three regression models during 10 years of follow-up in the study population

**Biomarker**	**Model I**			**Model II**			**Model III**		
Hazard ratio	95% CI	*P* value	Hazard ratio	95% CI	*P* value	Hazard ratio	95% CI	*P* value
Chromogranin A screen>median	1.53	1.06–2.20	0.02	1.34	0.92–1.94	0.12	1.27	0.88–1.84	0.21
Chromogranin A confirm>median	2.15	1.50–3.08	<0.0001	1.77	1.21–2.60	0.003	1.66	1.13–2.45	0.01
NT-proBNP>median	–	–	–	–	–	–	2.84	1.85–4.36	<0.0001

Model I, univariate analysis; model II, model adjusted for hypertension, ischemic heart disease, eGFR <60 ml/min, male gender, smoking habit, peripheral edema, rales, age >80 years, Hb <120 g/l; model III, all clinical variables from model II +4th quartile of NT-proBNP. Median of chromogranin A screen test: 65 pmol/l; median of chromogranin A confirm test: 531 pmol/l; median of NT-proBNP: 220 pmol/l. Each of the two biomarkers has been evaluated one at a time in the different models. NT-proBNP evaluation illustrated in model III is obtained from the analysis including chromogranin A confirm test.
